# Supplementary material for: An apical Phe-His pair defines the Orai1-coupling site and its occlusion within STIM1
Source: Nat Commun. 2023 Oct 30;14:6921. doi: 10.1038/s41467-023-42254-x (PMC10616141; doi:10.1038/s41467-023-42254-x)
Supplement: Supplementary file 1 — Supplementary Information [file 41467_2023_42254_MOESM1_ESM.pdf]

Supplementary Information

An apical Phe-His pair defines the Orai1-coupling site and its occlusion within STIM1

Yandong Zhou[1,a], Michelle R. Jennette[1*], Guolin Ma[2], Sarah A. Kazzaz[1], James H. Baraniak[1], Robert M. Nwokonko[3], Mallary L. Groff[1], Marcela Velasquez-Reynel[1], Yun Huang[4], Youjun Wang[5] and Donald L. Gill[1,a,b]*

[1] Department of Cellular and Molecular Physiology, The Pennsylvania State University College of Medicine, Hershey, PA 17033

[2] Institute of Biosciences and Technology, College of Medicine, Texas A&M University, Houston, TX 77030, USA

[3] Department of Molecular and Cellular Physiology, Stanford University School of Medicine, Stanford, CA, USA

[4] Center for Epigenetics and Disease Prevention, Institute of Biosciences and Technology, Texas A&M University, Houston, TX, 77030, USA

[5] Beijing Key Laboratory of Gene Resources and Molecular Development College of Life Sciences, Beijing Normal University, Beijing 100875, P.R. China

[a] To whom correspondence should be addressed: dongill@psu.edu or zhouyd@psu.edu

[b] Lead contact

* These authors contributed equally to the work

Supplementary Table 1. Key resources and reagents used in study

Reagent or resource	Source	Identifier
**Chemicals**		
ionomycin	Sigma-Aldrich	Cat#I9657
2-Aminoethoxydiphenyl borate (2-APB)	Millipore-Sigma	#D9754-10G
Fura-2 AM	Invitrogen	# F1201
**Cell Lines**		
Parental HEK293	ATCC	CRL-1573
HEK-S1S2dKO	Baraniak et al., 2021	N/A
HEK-S1S2dKO-O1CFP	Baraniak et al., 2021	N/A
**Software**		
SlideBook 6.0	Intelligent Imaging Innovations, Inc.	https://www.intelligent-imaging.com/slidebook
GraphPad Prism 9.0	GraphPad Prism	https://www.graphpad.com/scientific-software/prism/
Image J	Image Processing and Analysis in Java	https://imagej.nih.gov/ij/
**Commercial Kits/Assays**		
QuikChange Lightning Site-Directed Mutagenesis Kit	Agilent	Cat No. 210518

Supplementary Table 2. Oligonucleotide sequences used for cloning

Cloning Primers		
**Recombinant DNA**	Forward 5'------>3'	Reverse 5'------>3'
STIM1-YFP-F391H	AAAAGAAGAGAAACACACTC CAT GGCACCTTCCACGTGGCCCAC	GTGGGCCACGTGGAAGGTGCC ATG GAGTGTGTTTCTCTTCTTTT
STIM1-YFP-H395F	ACACACTCTTTGGCACCTTC TTC GTGGCCCACAGCTCTTCCCTGG	CCAGGGAAGAGCTGTGGGCCAC GAA GAAGGTGCCAAAGAGTGTGT
STIM1-YFP-F391H-H395F	AAAAGAAGAGAAACACACTC CAT GGCACCTTCTTCGTGGCCCAC	GTGGGCCACGAAGAAGGTGCC ATG GAGTGTGTTTCTCTTCTTTT
STIM1-YFP-F394H	GAGAAACACACTCTTTGGCACC CAC CACGTGGCCCACAGCTCTTCC	GGAAGAGCTGTGGGCCACGTG GTG GGTGCCAAAGAGTGTGTTTCTC
STIM1-YFP-H398F	GGC ACC TTC CAC GTG GCC TTC AGC TCT TCC CTG GAT GAT G	CAT CAT CCA GGG AAG AGC TGA AGG CCA CGT GGA AGG TGC C
STIM1-YFP-F394H-H398F	GGC ACC CAC CAC GTG GCC TTC AGC TCT TCC CTG GAT GAT G	CATCATCCAGGGAAGAGC TGA AGGCCACGTGGTGGGTGCC
STIM1-YFP-H398A	GGC ACC TTC CAC GTG GCC GCC AGC TCT TCC CTG GAT GAT G	CAT CAT CCA GGG AAG AGC TGG C GG CCA CGT GGA AGG TGC C

Continued

STIM1-YFP-H398G	GGCACCTTCCACGTGGCCGGCAGCTCTTCCCTG GATGATG	CATCATCCAGGGAAGAGCTGCCGGCCACGTGGA AGGTGCC
STIM1-YFP-H398D	GGCACCTTCCACGTGGCCGACAGCTCTTCCCTG GATGATG	CATCATCCAGGGAAGAGCTGTCGGCCACGTGGA AGGTGCC
STIM1-YFP-H398K	GGCACCTTCCACGTGGCC AAGAGCTCTTCCCTG GATGATG	CATCATCCAGGGAAGAGCTCTTGGCCACGTGGA AGGTGCC
STIM1-YFP-H398R	GGCACCTTCCACGTGGCCAGAAGCTCTTCCCTG GATGATG	CATCATCCAGGGAAGAGCTTCTGGCCACGTGGAAG GTGCC
STIM1-YFP-H398W	GGCACCTTCCACGTGGCCTGGAGCTCTTCCCTG GATGATG	CATCATCCAGGGAAGAGCTCCAGGCCACGTGGAAG GTGCC
STIM1-YFP-F394D	GAGAAACACACTCTTTGGCACC GAC CACGTGGCCCACAGCTCTTCC	GGAAGAGCTGTGGGCCACGTG GTC GGTGCCAAAGAGTGTGTTTCTC
STIM1-YFP-F394K	GAGAAACACACTCTTTGGCACC AAG CACGTGGCCCACAGCTCTTCC	GGAAGAGCTGTGGGCCACGTG CTT GGTGCCAAAGAGTGTGTTTCTC
STIM1-YFP-F394R	GAGAAACACACTCTTTGGCACC AGA CACGTGGCCCACAGCTCTTCC	GGAAGAGCTGTGGGCCACGTG TCT GGTGCCAAAGAGTGTGTTTCTC
STIM1-YFP-F394L	GAGAAACACACTCTTTGGCACC CTC CACGTGGCCCACAGCTCTTCC	GGAAGAGCTGTGGGCCACGTGGAGGGTGCCAAAGA GTGTGTTTCTC
STIM1-YFP-F394E	GAGAAACACACTCTTTGGCACC GAA CACGTGGCCCACAGCTCTTCC	GGAAGAGCTGTGGGCCACGTG TTC GGTGCCAAAGAGTGTGTTTCTC
STIM1-YFP F394 del	GAGAAACACACTCTTTGGCACC CACGTGGCCCACAGCTCTTCC	GGAAGAGCTGTGGGCCACGTG GGTGCCAAAGAGTGTGTTTCTC
STIM1-YFP H398 del	CTTTGGCACCTTCCACGTGGCC AGCTCTTCCCTGGATGATGTAG	CTACATCATCCAGGGAAGAGCT GGCCACGTGGAAGGTGCCAAAG
STIM1-YFP H395A	CACACTCTTTGGCACCTTC GCA GTGGCCCACAGCTCTTCC	GGAAGAGCTGTGGGCCAC TGC GAAGGTGCCAAAGAGTGTG
STIM1-YFP H395 del	CACACTCTTTGGCACCTTC GTGGCCCACAGCTCTTCC	GGAAGAGCTGTGGGCCAC GAAGGTGCCAAAGAGTGTG
STIM1-YFP 395-396 del	CACACTCTTTGGCACCTTC GCCCACAGCTCTTCCCTGGATG	CATCCAGGGAAGAGCTG TGGGCGAAGGTGCCAAAGAGTGTG
STIM1-YFP 396-397 del	GAAGAGAAACACACTCTTTGGC CACGTGGCCCACAGCTCTTCCC	GGGAAGAGCTGTGGGCCACGT GGCCAAAGAGTGTGTTTCTCTTC
STIM1-YFP Sα2 del	GAAGAGAAACACACTCTTTGGCAGCTCTTCCCTGGA TGATGTAGATC	GATCTACATCATCCAGGGAAGAGCTGCCAAAGAGTG TGTTTCTCTTC
STIM1-YFP 397-400 del	CTCTTTGGCACCTTCCACGTGTCCCTGGATGATGTA GATC	GATCTACATCATCCAGGGACACGTGGAAGGTGCCAA AGAG
STIM1-YFP-T393E	AGAGAAACACACTCTTTGGC GAA TTCCACGTGGCCCACAGCTCTTC	GAAGAGCTGTGGGCCACGTGGAA TTC GCCAAAGAGTGTGTTTCTCT
STIM1-YFP-T393K	AGAGAAACACACTCTTTGGC AAG TTCCACGTGGCCCACAGCTCTTC	GAAGAGCTGTGGGCCACGTGGAA CTT GCCAAAGAGTGTGTTTCTCT
STIM1-YFP-H395E	CACACTCTTTGGCACCTTC GAA GTGGCCCACAGCTCTTCC	GGAAGAGCTGTGGGCCAC TTC GAAGGTGCCAAAGAGTGTG

Continued

STIM1-YFP-H395K	CACACTCTTTGGCACCTTC AAG GTGGCCCACAGCTCTTCC	GGAAGAGCTGTGGGCCACCTTGAAGGTGCCA AAGAGTGTG
STIM1-YFP-D76A	ACATCCACAAACTGATGGAC GCT GATGCCAATGGTGATGTGGATG	CATCCACATCACCATTGGCATC AGC GTCCATCAGTTTGTGGATGT
STIM1-YFP-D76A-F394D	GAGAAACACACTCTTTGGCACC GAC CACGTGGCCCACAGCTCTTCC	GGAAGAGCTGTGGGCCACGTG GTC GGTGCCAAAGAGTGTGTTTCTC
STIM1-YFP-R304W	GCCCAGCGGCTGAAGGAGCTG TGG GAGGGTACTGAGAATGAGCGG	CCGCTCATTCTCAGTACCCTC CCA CAGCTCCTTCAGCCGCTGGGC
STIM1-YFP-K238E	CTATATCCAGAACCGTTACTCC GAG GAGCACATGAAGAAGATGATG	CATCATCTTCTTCATGTGCTC CTC GGAGTAACGGTTCTGGATATAG
STIM1-YFP-K238E-K242E-K243E	CGTTACTCCGAGGAGCACATG GAA GAA ATGATGAAGGACTTGGAGGGG	CCCCTCCAAGTCCTTCATCATTTCTTCCATGT GCTCCTCGGAGTAACG

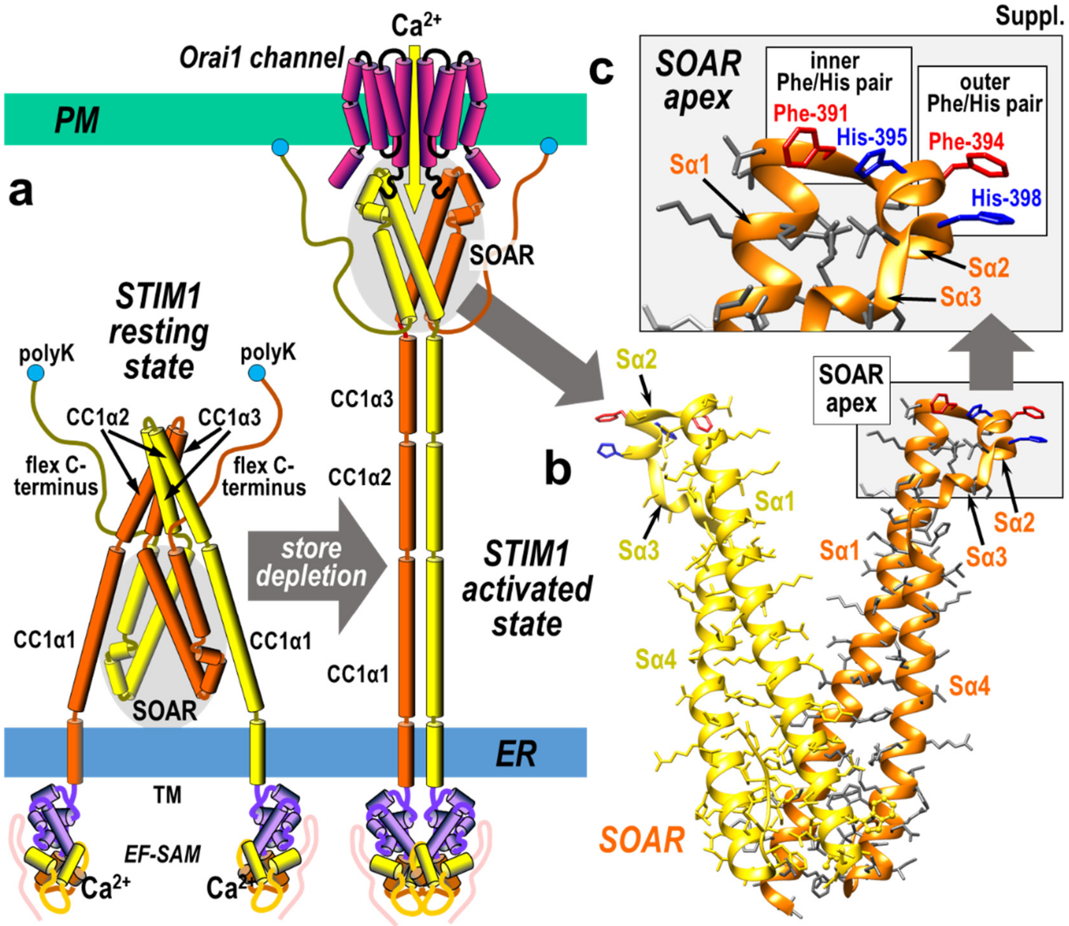

Supplementary Figure 1 | The structure and function of STIM1. **a** The resting state of the STIM1 dimer. SOAR is occluded within a "clamped" state through "domain-swapping" inter-dimer interactions between the CC1α1 helices and the CC3 coil of SOAR. TM, transmembrane domain; PolyK, poly-lysine C-terminus. After Ca^{2+} is depleted in the ER, the distant luminal EF-SAM domains associate, causing the TM and proximal CC1α1 helices to move together resulting in SOAR being "squeezed" and hence expelled and to begin to flip-out to the rear of the STIM1 protein. The CC1α1 and CC1α2 helices continue to bind together extending the length of STIM1, SOAR continues to flip-out, and the flexible C-termini are able to reach toward the PM, where their poly-lysine tails can associate with acidic phospholipids in the PM, tethering STIM1 to the PM. The three CC1 helices (CC1α1, CC12α2, and CC1α3) have fully bound and "zipped" together, giving STIM1 its fully extended conformation, which allows SOAR to bind to and activate Orai channels in the PM, permitting Ca^{2+} to enter and generate "store-operated" Ca^{2+} signals. **b** Structure of SOAR (STIM-Orai Activating Region)[40]: SOAR comprises 4 helices: Sα1, Sα2, Sα3 and Sα4. The 6-amino acid Sα2 helix (393-398) is located at the "apex" of SOAR and is crucial for Orai channel binding and activation. **c** Enlarged detail of the apical region shown in the gray rectangle in (**b**) includes two Phe-His pairs as shown, an "inner pair" and an "outer pair". (SOAR structure[40] was derived from PDB ID: 3TEQ).

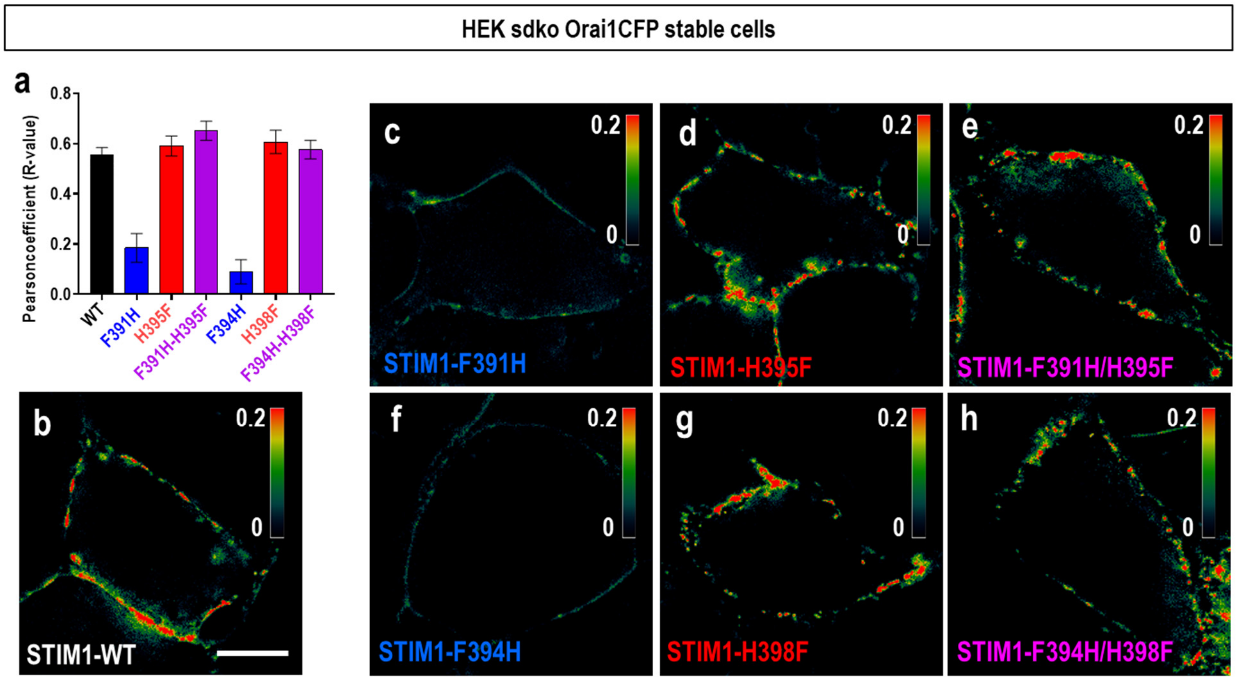

Supplementary Figure 2 | Pearson's correlation analysis and FRET images of data from Fig. 1. **a** Pearson's correlation coefficient (R-value) analysis was performed to assess the co-localization between Orai1-CFP and STIM1-YFP mutants compared to wild-type (WT) after treatment with 2.5 μM ionomycin, as depicted in Fig. 1a and 1f. Analysis was undertaken on multiple cells combined from 3 or more independent experiments. Cells numbers were: STIM1-WT (n=38), STIM1-F391H (n=39), STIM1-H395F (n=23), STIM1-F391H/H395F (n=27), STIM1-F394H (n=31), STIM1-H398F (n=20), STIM1-F394H/H398F (n=18). Data are means ± SEM. **b-h** FRET images are of single cells from the experiments shown in Fig. 1b and g, corresponding to STIM1-WT (**b**), STIM1-F391H (**c**), STIM1-H395F (**d**), STIM1-F391H/H398F (**e**), STIM1-F394H (**f**), STIM1-H398F (**g**), and STIM1-F394H/H398F (**h**). Scale bar: 10μm.

Suppl. Fig. 3

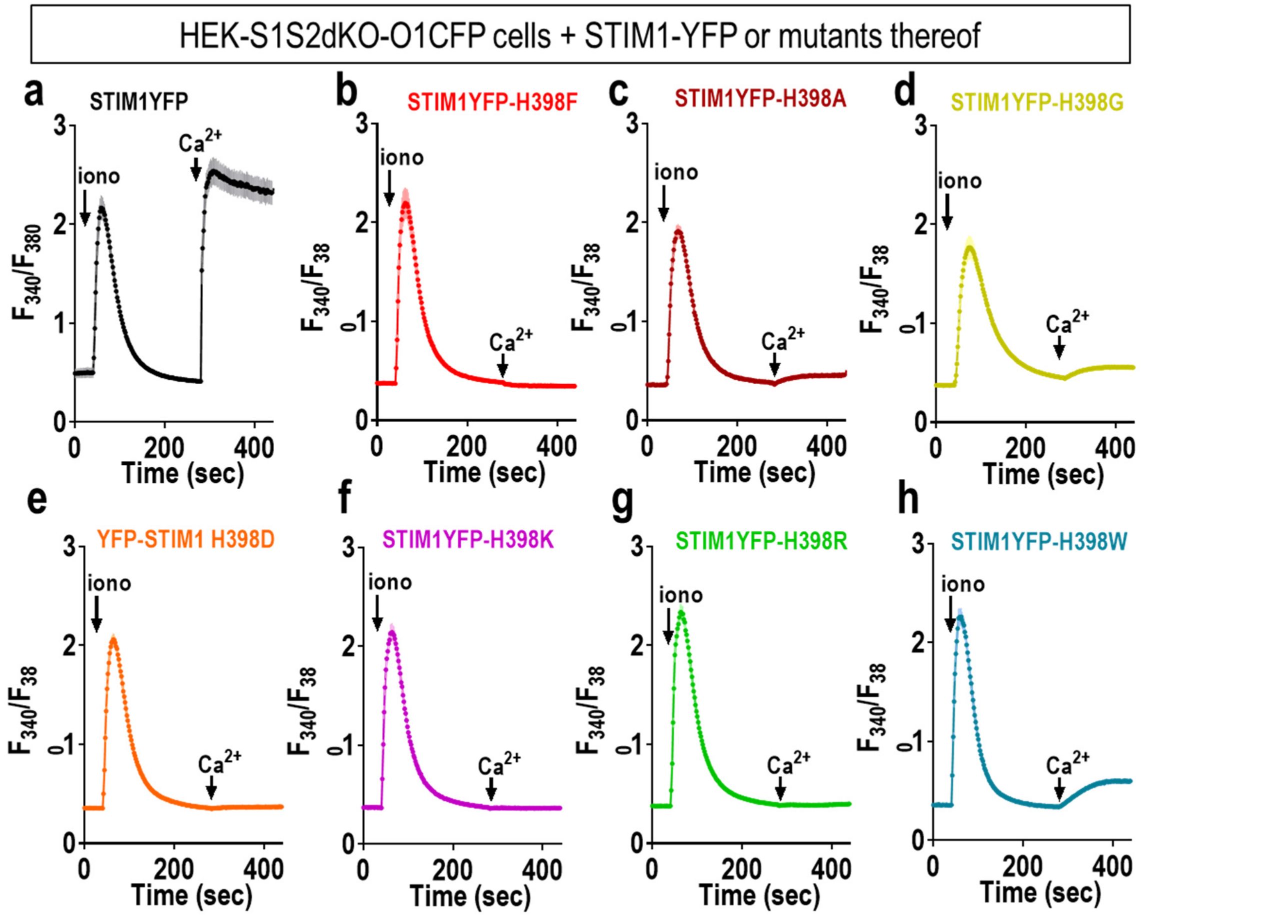

Supplementary Figure 3 │ Under store-replete conditions, HEK-S1S2dKO-O1CFP cells expressing STIM1-YFP-H398X mutations show little or no SOCE. Fura-2 Ca^{2+} responses in store-replete HEK-S1S2dKO-O1CFP cells expressing, **a** STIM1-YFP-WT (n=36), **b** STIM1-YFP-H398F (n=10), **c** STIM1-YFP-H398A (n=34), **d** STIM1-YFP-H398G (n=28), **e** STIM1-YFP-H398D (n=37), **f** STIM1-YFP-H398K (n=22), **g** STIM1-YFP-H398R (n=40), **h** STIM1-YFP-H398W (n=33). Statistics are presented in Fig. 3a.

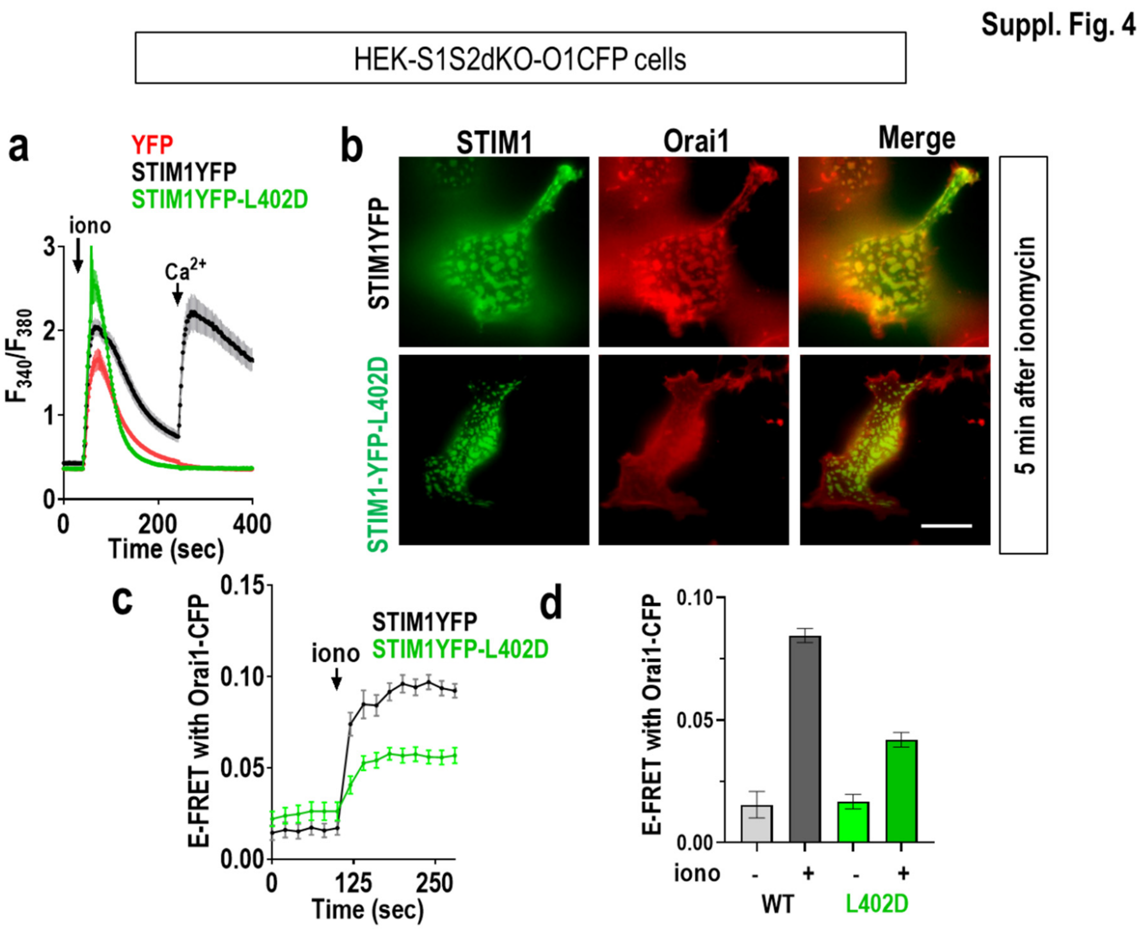

Supplementary Figure 4 │ The L402 mutation in the STIM1 Sα3 domain within SOAR , inhibits both STIM1-induced Ca^{2+} entry and E-FRET between YFP-STIM1 and with Orai1-CFP. a Fura-2 Ca^{2+} responses in store-replete HEK-S1S2dKO-O1CFP cells expressing STIM1-YFP WT (black, n=22), or STIM1-YFP-L402D (green, n=21), or YFP alone (red, n=26). **b** High-resolution imaging of the ER-PM interface in HEK-S1S2dKO-O1CFP cells transiently expressing STIM1-YFP WT or STIM1-YFP-L402D after 2.5 μM ionomycin treatment. Scale bar = 10μm. **c** E-FRET between Orai1-CFP and STIM1-YFP-WT (n=19) or STIM1-YFP-L402D (n=17) expressed in HEK-S1S2dKO-O1CFP cells as in (**a**). **d** Summary of E-FRET values before and after store-depletion for the STIM1 mutants shown in (**c**), STIM1-YFP WT (n=123), or STIM1-YFP-L402D (n=97). Values are means ± SEM from of three independent experiments, and the summary is change in E-FRET for the combined results for 4 experiments.

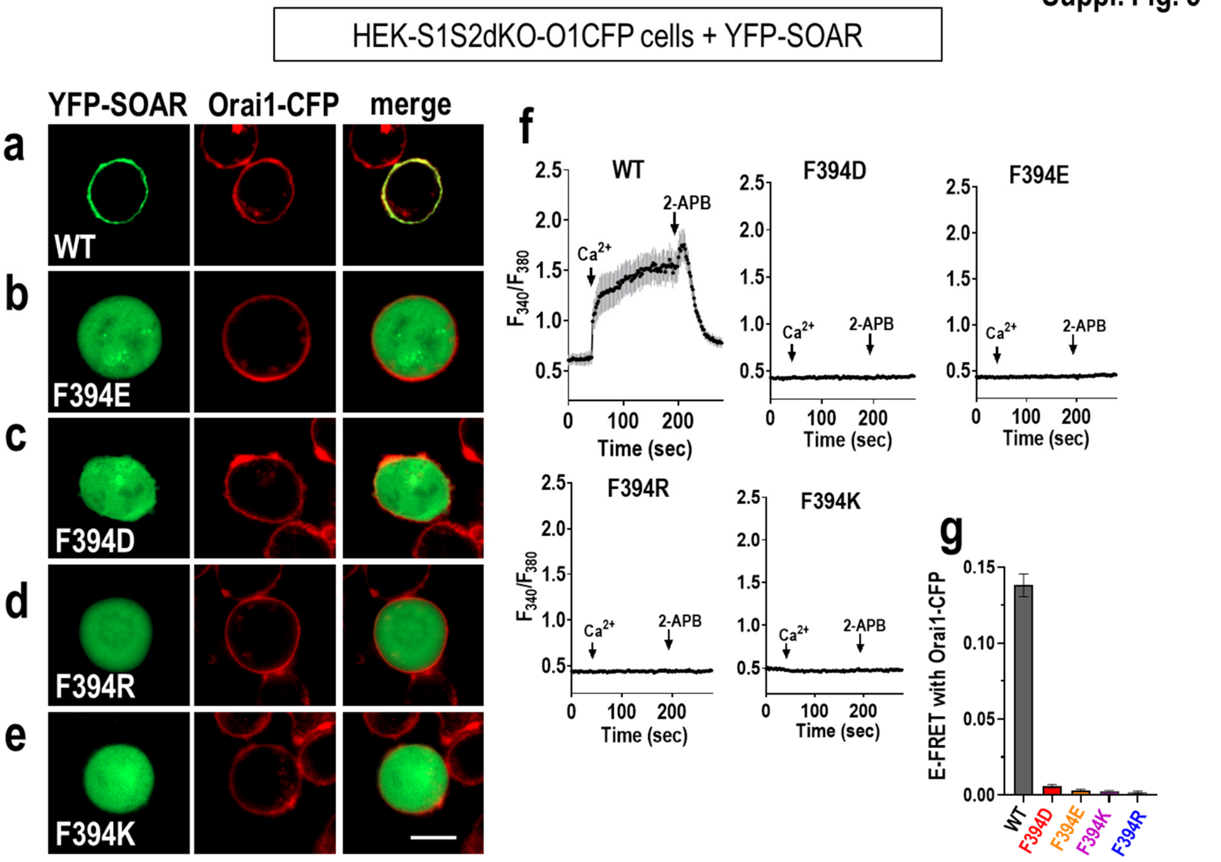

Supplementary Figure 5 │ Addition of positive or negative charged headgroups to replace F394 in expressed SOAR constructs, blocks Orai1-mediated Ca^{2+} entry and E-FRET with Orai1. SOAR constructs were transiently expressed in HEK-S1S2dKO-O1CFP cells. **a** The YFP-labeled wild-type SOAR (YFP-SOAR) was exclusively PM localized and its distribution was superimposable with Orai1. **b-e** In contrast, the YFP-tagged F394E, F394D, F394R or F394K mutants within SOAR were exclusively cytosolic. Scale bar = 10µm. **f** Fura-2 ratiometric Ca^{2+} responses in HEK-S1S2dKO-O1CFP cells transiently expressing similar levels of YFP-SOAR (n=15) or YFP-SOAR with either F394E (n=32), F394D (n=40), F394R (n=32) and F394K (n=35) mutations. The mutations all induced no measurable constitutive Ca^{2+} entry was measured in nominally Ca^{2+}-free medium and after addition of 1 mM Ca^{2+} (arrow). **g** Near-PM values of E-FRET measured in HEK-S1S2dKO-Orai1CFP stable cells between Orai1-CFP and transiently expressed YFP-SOAR WT (black, n=101), YFP-SOAR-F394D (blue, n=85), YFP-SOAR-F394E (purple, n=141), YFP-SOAR-F394K (green, n=119) or YFP-SOAR-F394R (red, n=155). Results are means ± SEM and representative of three independent repeats.

Suppl. Fig. 6

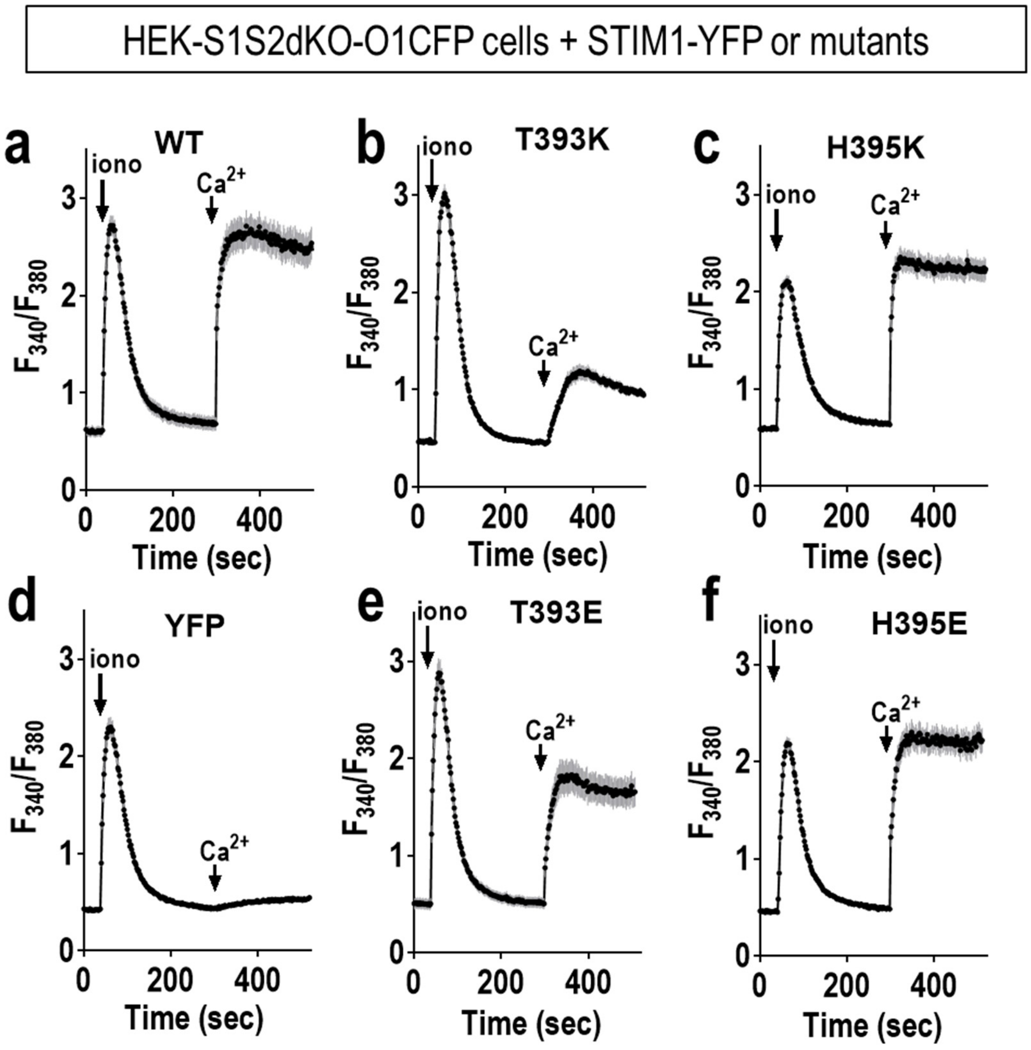

Supplementary Figure 6 │ Charged amino acids introduced into adjacent positions to F394 in STIM1 do not abolish SOCE. STIM1-YFP-WT, or the STIM1-YFP mutants shown or YFP negative control, were transiently expressed in HEK-S1S2dKO-O1CFP cells. Cytosolic Ca^{2+} signals measured by fura-2 ratiometric Ca^{2+} imaging in cells transiently expressing, **(a)** STIM1-YFP-WT (n=34), **(b)** STIM1-YFP-T393K (n=37), **(c)** STIM1-YFP-H395K (n=32), **(d)** YFP alone (n=22), **(e)** STIM1-YFP-T393E (n=36), or **(f)** STIM1-YFP-H395E (n=37). Results are typical of three separate experiments.

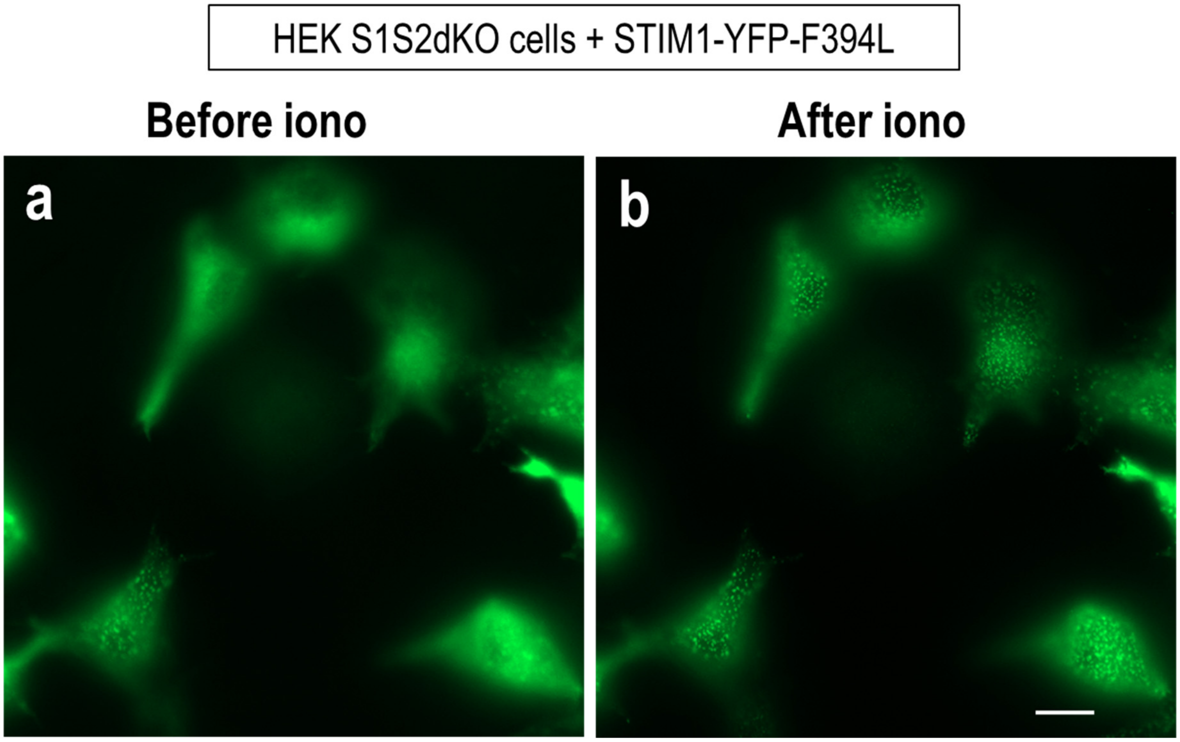

Supplementary Figure 7│The STIM1-YFP-F394L in STIM1 is partially within ER-PM junction puncta but still undergoes normal further activation after ER store-depletion. High-resolution imaging of the ER-PM interface in HEK-S1S2dKO cells transiently expressing STIM1-YFP-F394L. **a** Before ionomycin treatment. **b** 5 min after 2.5uM ionomycin treatment. Scale bar = 10μm.

Suppl. Fig. 8

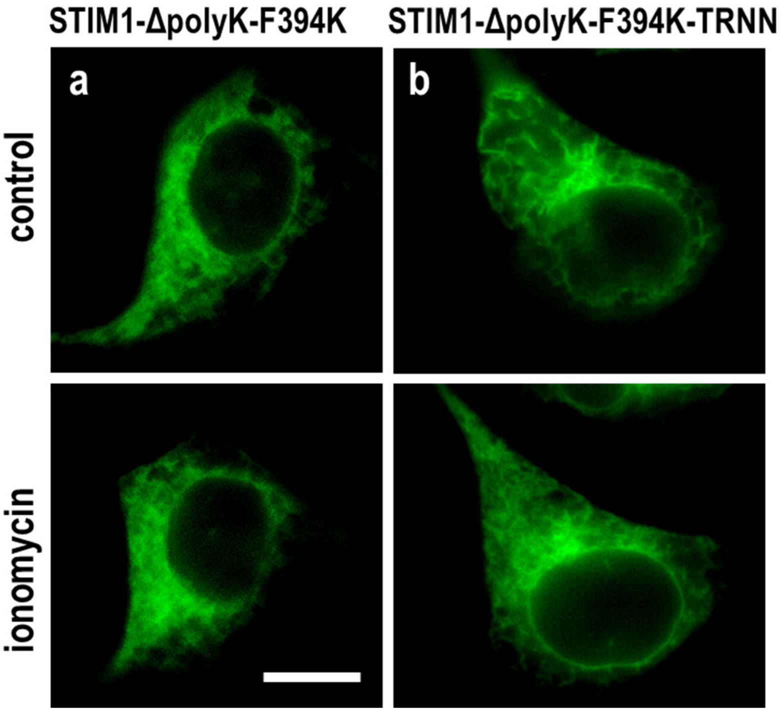

Supplementary Figure 8│ Deletion of the STIM1 EB-1-binding TRIP motif, does not rescue the inhibition of STIM1-F394K puncta due to poly-K deletion. The EB-1-binding TRIP motif (residues 642-645) can stabilize the inactive folded state of STIM1 by enhancing its interaction with microtubules in the vicinity of the ER. Elimination of the TRIP motif promotes the ability of STIM1 to translocate into ER-PM junctions[1]. We tested the construct in which the STIM1-F394K mutation was combined with ΔpolyK (**a**), and compared this construct with one containing the same two mutations together with the STIM1 "TRNN" mutation to delete the TRIP motif (**b**). Scale bar shown (10 μm) is for all cells. Cells were imaged before (top) or 5 min after (lower) ionomycin addition. The ΔpolyK deletion completely prevents the constitutively punctal STIM1-F394K mutation to relocate into puncta. The "TRNN" mutation did not rescue the defect caused by the ΔpolyK that prevents the F394K mutation-driven movement into puncta. Thus, the GoF TRNN mutation that causes constitutive activation of STIM1 cannot overcome the lack of attachment of STIM1-F394K to the PM as a result of removing the poly-K domain.

Suppl. Fig. 9

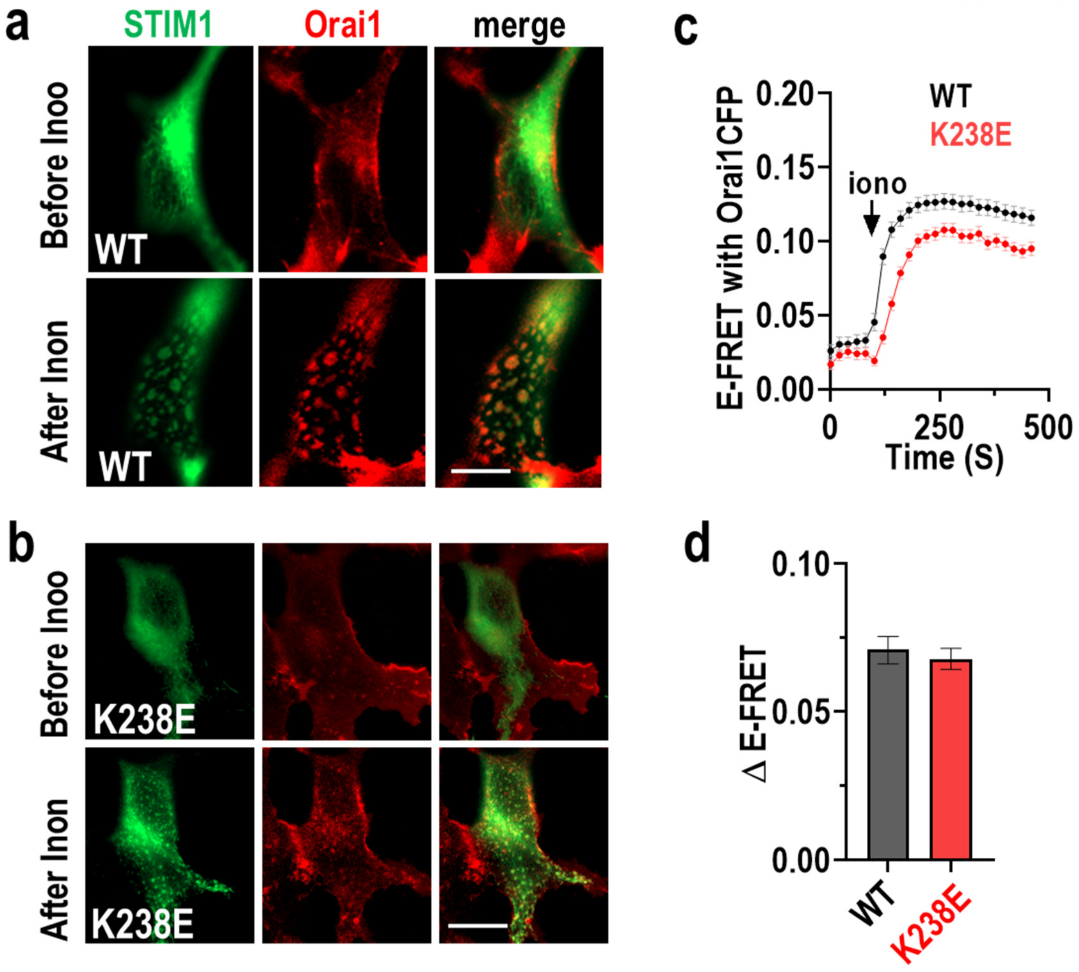

Supplementary Figure 9│ The K238E mutation does not significantly alter store-induced unclamping of STIM1 or its interaction with Orai1 channels. a-b High-resolution imaging of the ER–PM interface in HEK-S1S2dKO-O1CFP cells transiently expressing (**a**) STIM1-YFP-WT, or (**b**) STIM1-YFP-K238E, before (top) and after (bottom) 2.5 µM ionomycin treatment. Scale bar = 10µm. **c** Time course of E-FRET interactions between Orai1-CFP co-expressed with full-length STIM1-YFP-WT (black, n=23)**,** or STIM1-YFP-K238E (red, n=48). **d** Summary data of E-FRET analyses in (**c**), WT (n=93), K238E (n=104). Results are means ± SEM and representative of three independent repeats.

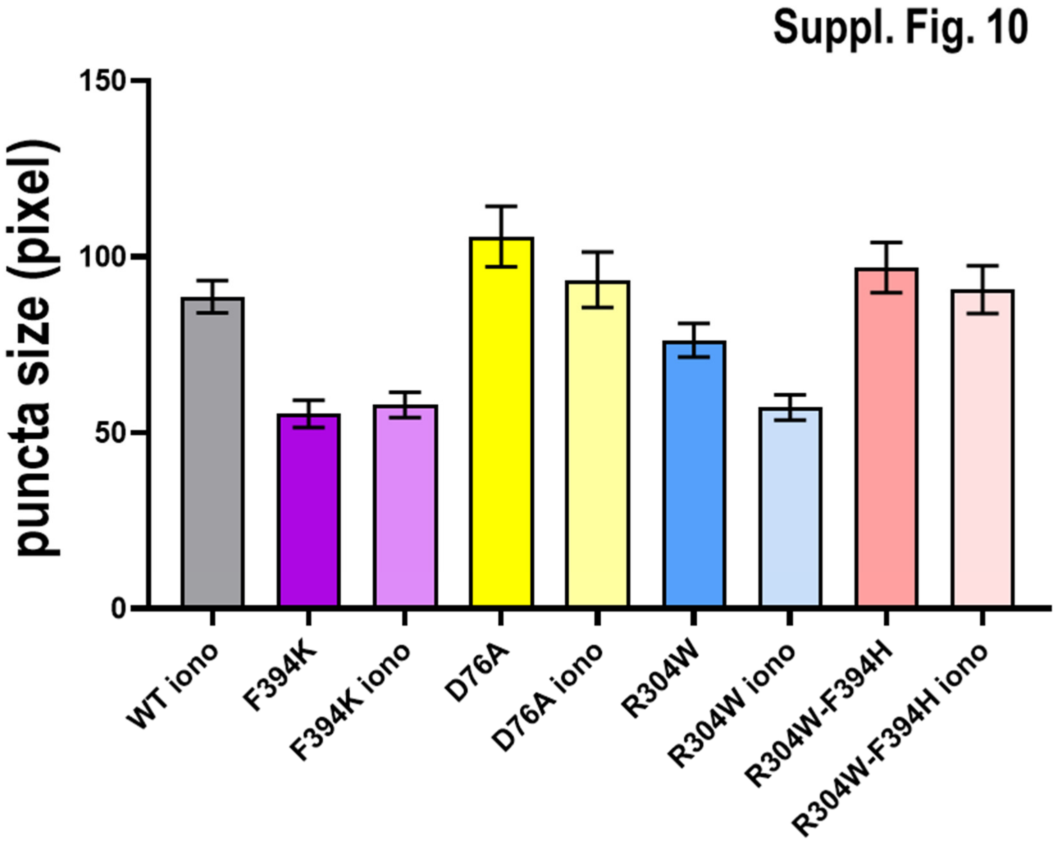

Supplementary Figure 10 │ Quantification of punctal size in STIM1 wild-type (WT) and the constitutively activated STIM1 mutants expressed in HEK-S1S2dKO cells, as shown in Fig. 6. The range of punctal size (pixels) was derived using ImageJ Watershed separation processing as described in Methods. Puncta number: STIM1-WT iono (ionomycin) (n=236 puncta from 4 cells), F394K (n=255 puncta from 5 cells), F394K iono (n=293 puncta from 5 cells), D76A (n=90 puncta from 3 cells), D76A iono (n=96 puncta from 3 cells), R304W (n=216 puncta from 4 cells), R304W iono (n=306 puncta from 4 cells), R304W-F394H (n=114 puncta from 3 cells), and R304W-F394H iono (n=113 puncta from 3 cells. Results shown are means ± SEM.

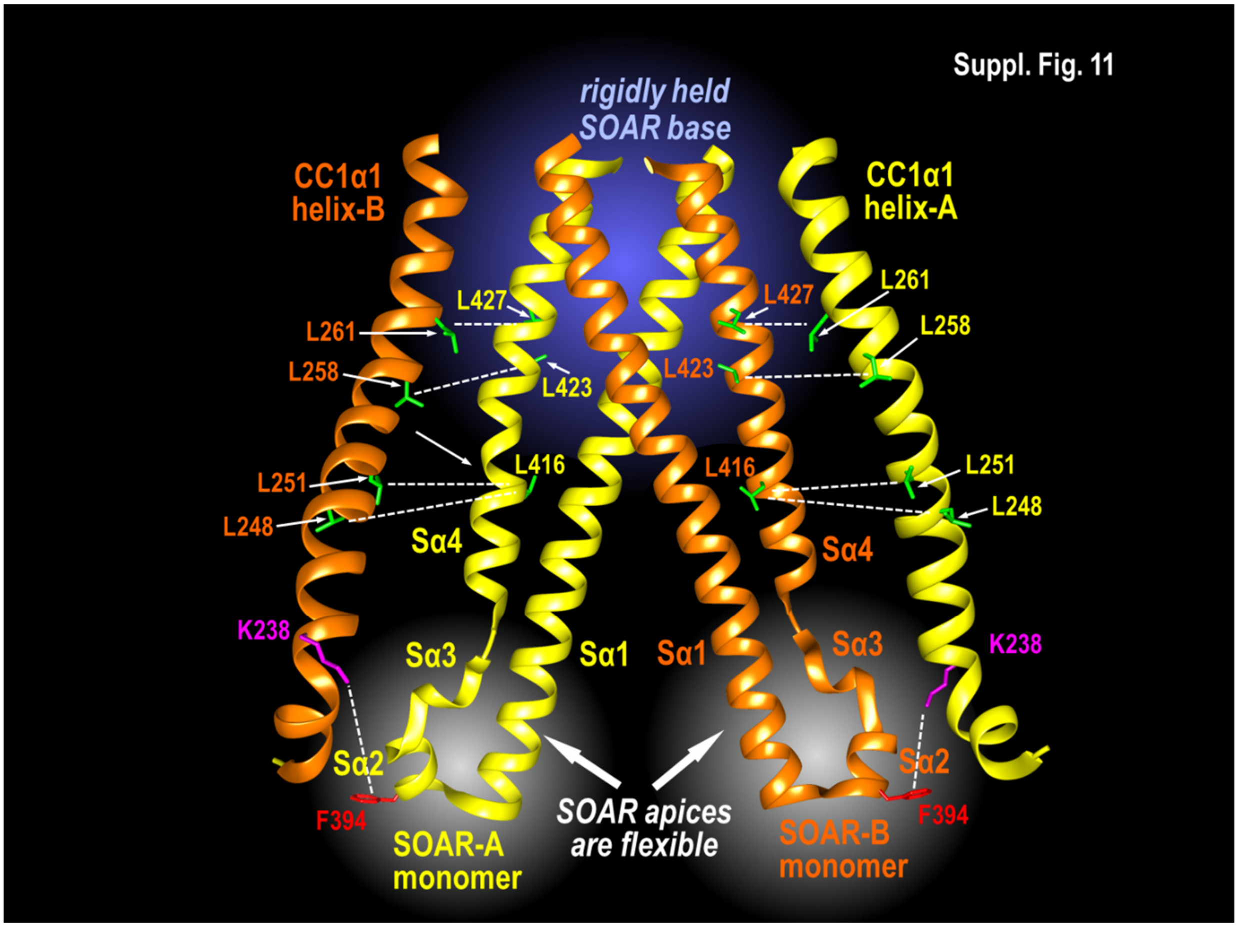

Supplementary Figure 11 │ Diagram depicting predicted clamping between the SOAR domain and CC1α1 helix of STIM1 under resting condition. The SOAR structure is from the crystal-derived structure of Yang et al[2] and CC1α1 is from the crystal structure of Cui et al [3]. The model is based on single molecule FRET studies of van Dorp et al [4] which indicate internal clamping within the STIM1 dimer occurs through parallel, inter-subunit, interactions between the CC1α1 helix and the Sα4 helix in the SOAR domain (see also Fig. 1). The predicted hydrophobic interactions between the L248, L251, L258, and L261 on CC1α1, and the L416, L423, L427 residues on Sα4, are represented by dotted lines. The diagram shows only an alignment of the interacting residues in the two helices, and is not a structural model. Also located, are the K238 and F394 residues in CC1α1 and Sα2, respectively. Based on the alignment of Leu residues shown, the K238 and F394 do not appear close enough to explain the charge repulsion postulated between K238 and the mutated F394K residue. However, whereas the SOAR base (at the top of the diagram) appears rigidly held in the STIM1 dimer, the SOAR apices (bottom of diagram) have some degree of flexibility within the holo-STIM protein [4], which would account for the close positioning of the 238 and 394 residues, and explain the complete unclamping of STIM1 induced by the F394K mutation.

Suppl. Fig. 12

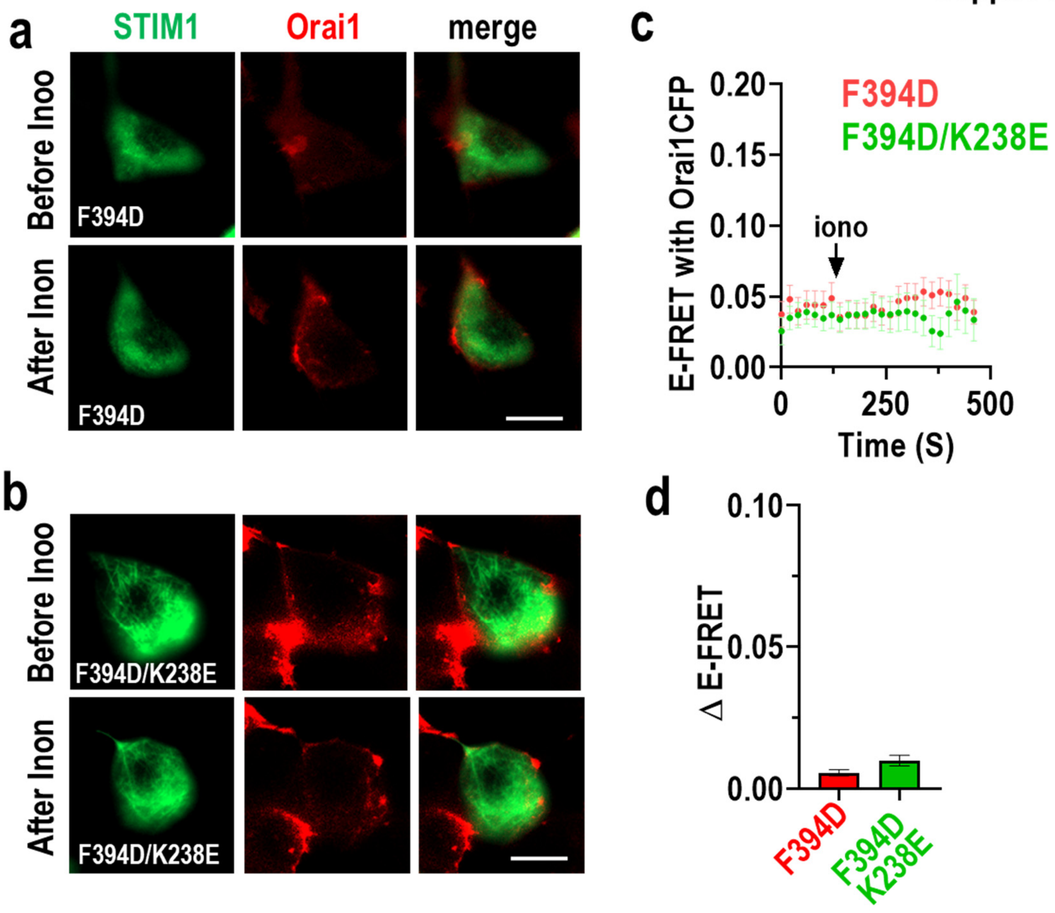

Supplementary Figure 12│ The K238E mutant does not alter the blocking phenotype of the F394D mutation within full length STIM1. a-b High-resolution imaging of the ER–PM interface in HEK-S1S2-dKO-O1CFP cells transiently expressing, (**a**) STIM1-YFP-F394D, or (**b**) STIM1-YFP-F394D/K238E, either before (top) or after (bottom) 2.5 μM ionomycin treatment. Scale bar = 10μm. **c** Time course of E-FRET interactions between Orai1-CFP co-expressed with full-length STIM1-YFP-F394D (red, n=9)**,** or STIM1-YFP-F394D/K238E (green, n=8). **d** Summary data for E-FRET analyses are shown in (**c**), WT (n=113), F394D/K238E (n=91). Results are means ± SEM and representative of three independent repeats.

Suppl. Fig. 13

```
Human STIM1        344   PEALQKWLQLTHEVEVQYYNIKKQNAEKQLLVAKEGAEKIKKKRNTLFG     392
Human STIM2        435   PDALQKWLQLTHEVEVQYYNIKRQNAEMQLAIAKDEAEKIKKKRSTVFG     483
Macaque STIM1      344   PEALQKWLQLTHEVEVQYYNIKKQNAEKQLLVAKEGAEKIKKKRNTLFG     392
Mouse STIM1        344   PEALQKWLQLTHEVEVQYYNIKKQNAERQLLVAKEGAEKIKKKRNTLFG     392
Drosophila STIM    410   PPQLQSWLQYTYELESKNHQKKRTSAEKQLQSAREACEKLRKKRSSLVG     458
C.elegans STIM     287   PLALQPLLRRTCENEMAFLEKQRQDCFKEMKEAIEMVDRLQKKQGSVLS     335

Human STIM1        393   TFHVAHS--SSLDDVDHKILTAKQALSEVTAALRERLHRWQQIEILCGFQIV  442
Human STIM2        484   TLHVAHS--SSLDEVDHKILEAKKALSELTTCLRERLFRWQQIEKICGFQIA  533
Macaque STIM1      393   TFHVAHS--SSLDDVDHKILTAKQALSEVTAALRERLHRWQQIEILCGFQIV  442
Mouse STIM1        393   TFHVAHS--SSLDDVDHKILTAKQALSEVTAALRERLHRWQQIEILCGFQIV  442
Drosophila STIM    459   AFVSTHG--KSIDDVDRSIVEARNALGDVTNELQERLHRWKQIETCLGLNIV  508
C.elegans STIM     336   SLKLATGAASTSDQVDSKIFALKSRMEKIHTLTRETQERWLQIESLCGFPLL  387
```

Supplementary Figure 13│ Comparison of SOAR sequences within STIM1 from human, macaque, and mouse, and the single STIM protein in *Drosophila* and *C. elegans*. Also shown is the sequence for human STIM2. The sequences reveal almost perfect alignment within the mammalian STIM1 proteins, and preservation of the critical apical outer Phe-His pair. Interestingly, the single STIM protein in *Drosophila*, also has a preserved apical outer Phe-His pair in the SOAR apex separated by three residues, even though the intervening amino acids are different. More interesting still, the *C. elegans* STIM sequence does not have any Phe-His pair in the SOAR apical region. This correlates well with the fact that recent data from Kim et al (2018) [5] reveal that the C. elegans STIM protein does not interact with or activate mammalian Orai1 channels. The C. elegans STIM protein does interact with the single *C. elegans* Orai channel. However, that channel is quite different to mammalian Orai channels. The TM4 nexus and TM4-extension sequences that are critical for STIM-binding in mammalian Orai channels [6,7], are very different in C elegans Orai.

Suppl. Fig. 14

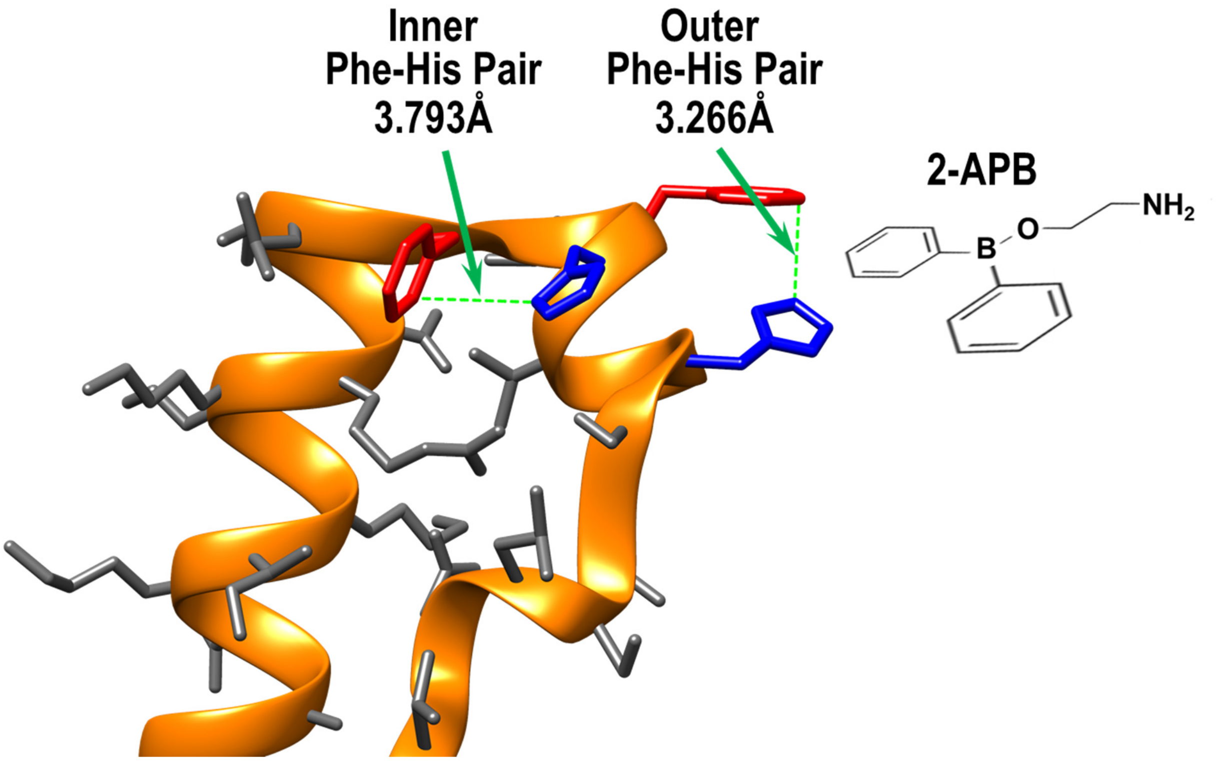

Supplementary Figure 14│ Structure and dimensions of the two Phe-His pairs in the SOAR apex of STIM1. The SOAR crystal structure [2] predicts the distances between aromatic rings in the inner and outer pair, are 3.79Å and 3.27Å, respectively. These distances and the offset angles between the aromatic rings are in keeping with structurally significant interactions within Phe-His pairs in many proteins [8-10]. Protonation of His greatly increases its π-stacking in aromatic pairs. Likely, the Phe-His pairs structurally restrain the partially-flexible SOAR apex in a conformation that assists both in clamping STIM1 in its resting state, and enhancing the conformational coupling to gate the Orai1 channel by activated STIM1. The structure of 2-APB is shown alongside the SOAR apex, and we hypothesize its interaction with Phe-His pairs or mutations thereof, may explain its effects on both STIM-Orai interactions and Orai channel gating.

Supplementary References

1. Kim, J. H. *et al.* The TAM-associated STIM1(I484R) mutation increases ORAI1 channel function due to a reduced STIM1 inactivation break and an absence of microtubule trapping. *Cell Calcium* **105**, 102615, doi:10.1016/j.ceca.2022.102615 (2022).
2. Yang, X., Jin, H., Cai, X., Li, S. & Shen, Y. Structural and mechanistic insights into the activation of Stromal interaction molecule 1 (STIM1). *Proc. Natl. Acad. Sci. U. S. A.* **109**, 5657-5662, doi:10.1073/pnas.1118947109 (2012).
3. Cui, B. *et al.* The inhibitory helix controls the intramolecular conformational switching of the C-terminus of STIM1. *PLoS One* **8**, e74735, doi:10.1371/journal.pone.0074735 (2013).
4. van Dorp, S. *et al.* Conformational dynamics of auto-inhibition in the ER calcium sensor STIM1. *Elife* **10**, doi:10.7554/eLife.66194 (2021).
5. Kim, K. M. *et al.* Distinct gating mechanism of SOC channel involving STIM-Orai coupling and an intramolecular interaction of Orai in Caenorhabditis elegans. *Proc. Natl. Acad. Sci. U. S. A.* **115**, E4623-E4632, doi:10.1073/pnas.1714986115 (2018).
6. Zhou, Y. *et al.* The STIM1-binding site nexus remotely controls Orai1 channel gating. *Nat Commun* **7**, 13725, doi:10.1038/ncomms13725 (2016).
7. Baraniak, J. H., Jr. *et al.* Orai channel C-terminal peptides are key modulators of STIM-Orai coupling and calcium signal generation. *Cell Rep* **35**, 109322, doi:10.1016/j.celrep.2021.109322 (2021).
8. Trachsel, M. A. *et al.* Modeling the Histidine-Phenylalanine Interaction. *J. Phys. Chem. B* **119**, 7778-7790, doi:10.1021/jp512766r (2015).
9. Bhattacharyya, R., Samanta, U. & Chakrabarti, P. Aromatic-aromatic interactions in and around alpha-helices. *Protein Eng.* **15**, 91-100, doi:10.1093/protein/15.2.91 (2002).
10. Chakrabarti, P. & Bhattacharyya, R. Geometry of nonbonded interactions involving planar groups in proteins. *Prog. Biophys. Mol. Biol.* **95**, 83-137, doi:10.1016/j.pbiomolbio.2007.03.016 (2007).
